# Automated Measurement of Cerebral Hemorrhagic Contusions and Outcomes After Traumatic Brain Injury in the TRACK-TBI Study

**DOI:** 10.1001/jamanetworkopen.2024.27772

**Published:** 2024-08-30

**Authors:** Samuel B. Snider, Nancy R. Temkin, Xiaoying Sun, Jacob L. Stubbs, Quinn J. Rademaker, Amy J. Markowitz, Eric S. Rosenthal, Ramon Diaz-Arrastia, Michael D. Fox, Geoffrey T. Manley, Sonia Jain, Brian L. Edlow

**Affiliations:** 1 Division of Neurocritical Care, Department of Neurology, Brigham and Women’s Hospital, Boston, Massachusetts; 2 Harvard Medical School, Boston, Massachusetts; 3 Department of Neurological Surgery, University of Washington, Seattle; 4 Department of Biostatistics, University of Washington, Seattle; 5 Biostatistics Research Center, Herbert Wertheim School of Public Health, University of California, San Diego; 6 Department of Medicine, University of British Columbia, Vancouver, British Columbia, Canada; 7 Department of Neurological Surgery, University of California, San Francisco; 8 Division of Clinical Neurophysiology, Department of Neurology, Massachusetts General Hospital, Boston; 9 Department of Neurology, University of Pennsylvania, Philadelphia; 10 Center for Brain Circuit Therapeutics, Departments of Neurology, Psychiatry, and Radiology, Brigham and Women’s Hospital, Boston, Massachusetts; 11 Berenson-Allen Center for Noninvasive Brain Stimulation, Department of Neurology, Beth Israel Deaconess Medical Center, Boston, Massachusetts; 12 Athinoula A. Martinos Center for Biomedical Imaging, Massachusetts General Hospital, Charlestown; 13 Brain and Spinal Cord Injury Center, Zuckerberg San Francisco General Hospital and Trauma Center, San Francisco, California; 14 Center for Neurotechnology and Neurorecovery, Department of Neurology, Massachusetts General Hospital, Boston

## Abstract

**Importance:**

Because withdrawal of life-sustaining therapy based on perceived poor prognosis is the most common cause of death after moderate or severe traumatic brain injury (TBI), the accuracy of clinical prognoses is directly associated with mortality. Although the location of brain injury is known to be important for determining recovery potential after TBI, the best available prognostic models, such as the International Mission for Prognosis and Analysis of Clinical Trials in TBI (IMPACT) score, do not currently incorporate brain injury location.

**Objective:**

To test whether automated measurement of cerebral hemorrhagic contusion size and location is associated with improved prognostic performance of the IMPACT score.

**Design, Setting, and Participants:**

This prognostic cohort study was performed in 18 US level 1 trauma centers between February 26, 2014, and August 8, 2018. Adult participants aged 17 years or older from the US-based Transforming Research and Clinical Knowledge in TBI (TRACK-TBI) study with moderate or severe TBI (Glasgow Coma Scale score 3-12) and contusions detected on brain computed tomography (CT) scans were included. The data analysis was performed between January 2023 and February 2024.

**Exposures:**

Labeled contusions detected on CT scans using Brain Lesion Analysis and Segmentation Tool for Computed Tomography (BLAST-CT), a validated artificial intelligence algorithm.

**Main Outcome and Measure:**

The primary outcome was a Glasgow Outcome Scale–Extended (GOSE) score of 4 or less at 6 months after injury. Whether frontal or temporal lobe contusion volumes improved the performance of the IMPACT score was tested using logistic regression and area under the receiver operating characteristic curve comparisons. Sparse canonical correlation analysis was used to generate a disability heat map to visualize the strongest brainwide associations with outcomes.

**Results:**

The cohort included 291 patients with moderate or severe TBI and contusions (mean [SD] age, 42 [18] years; 221 [76%] male; median [IQR] emergency department arrival Glasgow Coma Scale score, 5 [3-10]). Only temporal contusion volumes improved the discrimination of the IMPACT score (area under the receiver operating characteristic curve, 0.86 vs 0.84; *P* = .03). The data-derived disability heat map of contusion locations showed that the strongest association with unfavorable outcomes was within the bilateral temporal and medial frontal lobes.

**Conclusions and Relevance:**

These findings suggest that CT-based automated contusion measurement may be an immediately translatable strategy for improving TBI prognostic models.

## Key Points

**Question:**

What is the association between automated measurement of hemorrhagic contusions detected on computed tomography scans and outcomes after traumatic brain injury (TBI)?

**Findings:**

In this prognostic cohort study of 291 patients with cerebral hemorrhagic contusions after TBI, automated measurement of contusion volume in the temporal, but not frontal, lobes was associated with improved performance of the criterion standard IMPACT prognostic model. The data-derived disability heat map highlighted a specific set of brain regions critical for determining outcomes.

**Meaning:**

These findings suggest that automated measurement of brain injury detected on computed tomography scans may be an immediately translatable tool to mechanistically understand and better predict disability outcomes after TBI.

## Teaser

This prognostic cohort study tests whether automated measurement of cerebral hemorrhagic contusion size and location is associated with improved clinical prognoses after traumatic brain injury (TBI).

## Introduction

Because withdrawal of life-sustaining therapy is the most common cause of death following moderate or severe traumatic brain injury (TBI),^1,2^ the accuracy of clinical prognoses is directly associated with mortality. Prognostic logistic regression models, such as the International Mission for Prognosis and Analysis of Clinical Trials in TBI (IMPACT) score,^3^ have been developed to assign a probability of 6-month mortality or unfavorable outcome. While these models have been externally validated,^4^ their calibration has been questioned,^5^ and they were not intended for use at the single-patient level. Furthermore, these models incorporate little information about the location of the underlying brain injury and have achieved limited clinical penetration.^6^

Hemorrhagic contusions (ie, injuries resulting from contact between brain parenchyma and the rough skull surface)^7^ are among the most common brain lesions observed after TBI.^8,9^ Very large contusions (>25 cm^3^) have been associated with worse outcomes,^10^ and their presence or absence is an element of the IMPACT score.^3^ However, it is unclear whether contusions in different brain locations have differential associations with outcomes and should be considered independently by prognostic models.

The recently developed and validated Brain Lesion Analysis and Segmentation Tool for Computed Tomography (BLAST-CT),^11^ an open-source, artificial intelligence–based tool to identify contusions on computed tomography (CT) scans for patients with TBI, creates new opportunities to investigate the prognostic utility of contusion volume and location. Taking a brain CT as input, BLAST-CT generates a multiclass label that includes cerebral parenchymal hemorrhage, cerebral edema, extraparenchymal hemorrhage, and intraventricular hemorrhage. These labels provide a simple means of quantifying the location and extent of brain injury apparent on CT scans. We applied this novel contusion-labeling algorithm to data from the US-based, 18-center Transforming Research and Clinical Knowledge in TBI (TRACK-TBI) study^12^ to test whether automated contusion measurement (1) identifies regional variation in the association between contusion volume and outcomes and (2) improves the best available TBI prognostic models.

## Methods

For this prognostic cohort study, the institutional review boards at each TRACK-TBI site provided approval, and participants or surrogates provided written informed consent (local oversight by the Mass General Brigham institutional review board). All studies have therefore been performed in accordance with the ethical standards of the Declaration of Helsinki.^13^ This report followed the Transparent Reporting of a Multivariable Prediction Model for Individual Prognosis or Diagnosis (TRIPOD) reporting guideline for prognostic studies.

### Study Cohort

TRACK-TBI is a prospective study of patients with acute TBI who presented to 1 of 18 US level 1 trauma centers and received a clinically indicated head CT scan between February 26, 2014, and August 8, 2018. Details of the overarching design of TRACK-TBI and 1-year outcomes have been published previously.^12,14,15^ Notably, participants with multisystem trauma that was expected to interfere with follow-up or outcome assessments were excluded. Within TRACK-TBI, we extracted a cohort of adults (aged ≥17 years) with moderate or severe TBI (Glasgow Coma Scale [GCS] score <13 on a scale of 3 [most impaired] to 15 [least impaired] at any point in the first 24 hours) with at least 1 CT scan within 48 hours of injury and nonzero BLAST-CT–measured contusion volume. We also collected demographic data, including sex, self-reported race (Black, White, other [American Indian, Asian, and Native Hawaiian or Other Pacific Islander]), self-reported ethnicity (Hispanic and non-Hispanic), years of education, cause of injury, hospital admission disposition, and history of TBI and/or psychiatric disorders. Race and ethnicity data were included as a descriptive variable and not used as a covariate in the statistical analysis.

### Primary Outcome

In our primary analyses, an unfavorable neurologic outcome was defined as a Glasgow Outcome Scale–Extended (GOSE) score of 4 or less at 6 months after injury, which encompasses death or severe disability with functional dependency. In TRACK-TBI, the GOSE is prospectively assessed by trained assessors as part of a comprehensive outcome battery. An outcome assessment specifically elicited the participant’s functional impairment attributable to the TBI and not impairment related to injuries in extracranial body systems. Large observational and randomized clinical trials of TBI have used 6-month GOSE scores of 3 or less (lower severe disability, eg, cannot be left unsupervised in the home for >8 hours)^14,16,17^ and GOSE scores of 4 or less (upper severe disability, eg, can be left unsupervised for 8-24 hours in the home or is dependent outside the home)^18,19,20,21^ as the primary outcome. We chose a GOSE score of 4 or less as our primary end point to match the outcome used when developing the IMPACT model.^3^ To ensure that our results did not depend on the specific cutoff score, we repeated analyses using a secondary outcome of a GOSE score of 3 or less, a cut point better aligned with family and caregiver assessments of an unfavorable outcome.^22^

### Brain CT Analysis

We analyzed the latest CT scan available within 48 hours of the earliest available scan because contusions commonly expand beyond their initial appearance^23^ and most expansions occur within 24 hours of the first CT scan.^24^ Scan acquisition parameters and scanners were not standardized across sites. We used the axial series with 5-mm slice separation when available or the 2- or 3-mm slice separation if not. We developed a custom pipeline using open-source tools to map hemorrhagic contusions detected on CT scans in a scalable fashion. All image processing was performed by a board-certified neurocritical care physician (S.B.S.) without access to outcomes data.

To generate a contusion label map for each participant, we used the recently described BLAST-CT algorithm.^11^ BLAST-CT is a convolutional neural network based on the DeepMedic architecture^25^ that has been trained and validated against criterion standard manual labels in diverse European and Indian TBI datasets. For an individual CT scan, BLAST-CT generates a multiclass label that includes parenchymal hemorrhage, parenchymal edema, intraventricular hemorrhage, and extraparenchymal hemorrhage (subdural and/or subarachnoid hemorrhage). Because contusions have both hyperdense (acute blood products) and hypodense (cytotoxic and vasogenic edema) components on a CT scan,^26,27^ we combined the parenchymal hemorrhage and edema labels to obtain a single binary contusion label. In a sensitivity analysis, we analyzed the hyperdense and hypodense components separately.

BLAST-CT has been previously validated.^11^ To confirm performance within the TRACK-TBI data, we correlated BLAST-CT labels to manually traced labels in a subset of 16 participants (eFigure 1 in Supplement 1). BLAST-CT and manual contusion label volumes were tightly correlated (*R* = 0.9; *P* < .001).

To enable atlas-based measurements of regional contusion volumes and voxelwise analyses, we registered CT scans into a common coordinate system. The CT scans were first skull stripped using ichseg,^28^ an R-based tool, and then registered to a 2-mm isotropic standard CT template^29^ using linear and nonlinear registration (ANTsSynQuick; University of Pennsylvania). Registrations were manually checked, and participants with grossly misaligned scans were excluded. We then performed a similar registration between CT template space and Montreal Neurological Institute (MNI) T1 1-mm template space to allow for the registration of MNI atlas labels^30^ onto individual participant’s CT scans.

For regional volumetric analysis, we registered MNI atlas labels onto each participant’s CT scan and computed the total volume of contusions overlapping each atlas label. For the voxelwise analysis, we used contusion labels registered into CT template space and down sampled to a 3-mm isotropic voxel size.

### Clinical Covariates

For each participant, we computed the IMPACT (core plus CT) score, defined as the probability of an unfavorable outcome using the original IMPACT model coefficients.^3,31^ The component predictors are prospectively entered into a TRACK-TBI database from each site and include age, GCS motor score (1-6, indicating best motor response), pupillary reactivity (both reacting, 1 reacting, and neither reacting), the Marshall CT Classification score (I [diffuse injury, no visible pathology] to VI [nonevacuated mass lesion]),^10^ the presence of traumatic subarachnoid hemorrhage or epidural hemorrhage on initial CT scan, and hypoxia and hypotension on admission. The IMPACT model has been repeatedly shown to produce a well-calibrated estimate of the probability of death or severe disability at 6 months after injury.^4^ We excluded participants with missing data for any component variable.

### Statistical Analysis

The data analysis was performed between January 2023 and February 2024. We assessed contusion volumes from the 2 most common locations (frontal and temporal lobes) and tested their independent associations with the outcome, adjusting for the IMPACT score. Regional volumes were categorized into absent, 2 cm^3^ or less, and more than 2 cm^3^ for analysis. A volume of 2 cm^3^ corresponded to approximately the 75th percentile of both frontal and temporal lobe contusion volumes. We compared the area under the receiver operating characteristic curve (AUROC) of multivariable logistic regression models, including IMPACT score (model 1), IMPACT plus frontal contusion volumes (model 2), IMPACT plus temporal contusion volumes (model 3), and IMPACT plus frontal and temporal contusion volumes (model 4) using Delong tests. An a priori significance level was set at *P* < .05, and all hypothesis tests were 2-sided.

We next sought to identify the brain locations most strongly associated with the outcome using multivariable lesion symptom mapping. We used sparse canonical correlation analysis^32^ as implemented in the LESYMAP package in R, version 1.4.1717,^33^ an approach with several advantages compared with mass univariable testing.^33,34,35^ Sparse canonical correlation analysis identifies a linear combination of voxels that collectively explain the most variance in outcomes. This analysis is performed using 4-fold cross-validation, iterating through combinations of weights in the training set that show the strongest correlation with the outcome in the test set. Weights are iteratively smoothed, spatially isolated voxels are set to 0, and maps with lower sparseness (more voxels) are penalized. The sparseness value that leads to the best predictive performance in the held-out fold is retained. The final set of weights are normalized from 0 to 1, with higher values indicating a stronger association with the outcome; weights below 10% of the maximum value are set to 0. We tested only voxels with a contusion in at least 5 participants, a standard threshold for voxelwise analyses.^36,37^ The resulting *P* value refers to the significance of the Pearson correlation with outcomes in the held-out testing fold using the weights from the final map (disability heat map). We ran this analysis using the primary outcome (GOSE score ≤4), as well as treating GOSE as a continuous measure. The final maps were registered to a high-resolution MNI template for visualization.^38^

## Results

### Cohort Characteristics

Of 2552 TRACK-TBI participants, 417 met the inclusion criteria. The analysis cohort included 291 participants with moderate or severe TBI (GCS score of 3-12), assessable CT scans, nonzero contusion volumes, all elements of the IMPACT score, and GOSE scores acquired at 6 months after injury (eFigure 2 in Supplement 1). The mean (SD) age of the participants was 42 (18) years, and 70 participants (24%) were female and 221 (76%) male. Of 285 participants with data on race, 37 (13%) identified as Black, 231 (81%) as White, and 17 (6%) as other. Of 286 participants with ethnicity data, 50 (17%) identified as Hispanic and 236 (83%) as non-Hispanic (Table 1). The median emergency department arrival GCS score was 5 (IQR, 3-10) (Table 1); 141 participants (48%) had an unfavorable outcome (GOSE score ≤4) at 6 months (eFigure 3 in Supplement 1), and 134 (46%) had a GOSE score of 3 or less. Participants with unfavorable outcomes were older and had a lower emergency department arrival GCS as well as other markers of a greater injury severity (Table 1). Compared with the analysis cohort, participants with missing IMPACT component variables (n = 126) had no differences in demographics, injury severities, or contusion volumes (eTable 1 in Supplement 1; Table 2) but a greater proportion of unfavorable outcomes (64% vs 48%; *P* = .003) (eFigure 3 in Supplement 1). Participants with missing 6-month GOSE scores were younger; more likely to be Black, other race, or Hispanic; had slight differences in injury mechanisms and initial GCS scores; and were less likely to be admitted to the intensive care unit (eTable 1 in Supplement 1).

**Table 1.** Cohort Characteristics

Characteristic	Total, No. (%)	Outcomes, No. (%)		*P* value
		Favorable (GOSE score 5-8)	Unfavorable (GOSE score 1-4)	
Age, mean (SD), y	42 (18)	35 (15)	49 (19)	<.001
Sex				
Female	70 (24)	33 (22)	37 (26)	.41
Male	221 (76)	117 (78)	104 (74)	
Race				
Black	37 (13)	20 (13)	17 (13)	.95
White	231 (81)	122 (81)	109 (81)	
Other^a^	17 (6)	8 (5)	9 (7)	
Total	285 (100)	150 (100)	135 (100)	
Ethnicity				
Hispanic	50 (17)	25 (17)	25 (18)	.76
Non-Hispanic	236 (83)	124 (83)	112 (82)	
Total	286 (100)	149 (100)	137 (100)	
Education, mean (SD), y	13 (2)	13 (2)	13 (3)	.11
Disposition				
ED discharge	1 (0)	1 (0)	0	.12
Hospital admission	4 (1)	4 (3)	0	
ICU admission	286 (98)	145 (97)	141 (100)	
Total	291 (100)	150 (100)	141 (100)	
Injury cause				
Road traffic incident	165 (56)	84 (57)	81 (57)	.84
Incidental fall	81 (28)	40 (27)	41 (30)	
Violence or assault	17 (7)	9 (6)	8 (6)	
Other	25 (9)	15 (10)	10 (7)	
Total	288 (100)	148 (100)	140 (100)	
Psychiatric history	69 (24)	37 (25)	32 (23)	.78
Prior TBI	41 (16)	21 (15)	120 (18)	.50
GCS score on ED arrival^b^				
13-15	36 (13)	26 (18)	10 (7)	.004
9-12	54 (19)	33 (23)	21 (15)	
3-8	191 (68)	86 (59)	105 (77)	
Total	281 (100)	145 (100)	136 (100)	
Median (IQR)	5 (3-10)	7 (3-11)	3 (3-8)	<.001
Initial CT abnormal finding^c^	276 (95)	137 (91)	139 (99)	.006
Marshall CT Classification score^c^^,^^d^				
I (no visible pathology)	15 (5)	13 (9)	2 (1)	<.001
II	113 (39)	74 (49)	39 (28)	
III-IV	30 (10)	16 (11)	14 (10)	
V-VI (evacuated or nonevacuated mass lesion)	133 (46)	47 (31)	86 (61)	
Total	291 (100)	150 (100)	141 (100)	
EDH^c^	56 (19)	36 (24)	20 (14)	.04
SAH^c^	239 (82)	108 (72)	131 (93)	<.001
GCS motor score^e^				
5 or 6	108 (37)	74 (49)	34 (24)	.001
4	34 (12)	20 (13)	14 (10)	
3	12 (4)	5 (3)	7 (5)	
2	16 (6)	5 (3)	11 (8)	
1	121 (42)	46 (31)	75 (53)	
Total	291 (100)	150 (100)	141 (100)	
Pupil reactivity				
Both reacted	206 (71)	124 (83)	82 (58)	<.001
1 Reacted	25 (9)	6 (4)	19 (13)	
Neither reacted	60 (21)	20 (13)	40 (28)	
Total	291 (100)	150 (100)	141 (100)	
Hypotension	32 (11)	11 (7)	21 (15)	.06
Hypoxia	41 (14)	16 (11)	25 (18)	.09

Abbreviations: CT, computed tomography; ED, emergency department; EDH, epidural hemorrhage; GCS, Glasgow Coma Scale; GOSE, Glasgow Outcome Scale–Extended; ICU, intensive care unit; SAH, subarachnoid hemorrhage; TBI, traumatic brain injury.

^a^ Included American Indian, Asian, and Native Hawaiian or Other Pacific Islander.

^b^ Higher scores indicate less impairment.

^c^ All variables in this table were taken from the initial CT scan.

^d^ Class I indicates no visible pathology; II, diffuse injury, cisterns visible; III, diffuse injury, cisterns compressed; IV, diffuse injury midline shift greater than 5 mm; V, any evacuated mass lesion; and VI, nonevacuated high or mixed-density lesion greater than 25 cm^3^.

^e^ Motor scores of 1 (no movement) to 6 (following commands).

**Table 2.** Contusion Volumes by Outcome

Volume percentile	Outcome			IMPACT variables		
	Favorable (GOSE score 5-8)	Unfavorable (GOSE score 1-4)	*P* value	Available	Missing	*P* value
**Total volume, cm^3^**						
25th	0.17	1.44	<.001	0.39	0.33	.25
50th	0.75	6.35		2.44	4.38	
75th	4.44	23.31		13.22	19.38	
**Frontal volume, cm^3^**						
25th	0	0.12	<.001	0.02	0.02	.22
50th	0.14	0.91		0.31	0.76	
75th	1.00	3.89		2.20	3.49	
**Temporal volume, cm^3^**						
25th	0	0.10	<.001	0	0.01	.27
50th	0.05	1.01		0.21	0.27	
75th	0.37	8.01		2.07	3.22	

Abbreviations: GOSE, Glasgow Outcome Scale–Extended; IMPACT, International Mission for Prognosis and Analysis of Clinical Trials in TBI.

### Regional Contusion Volumes

**Figure 1.**
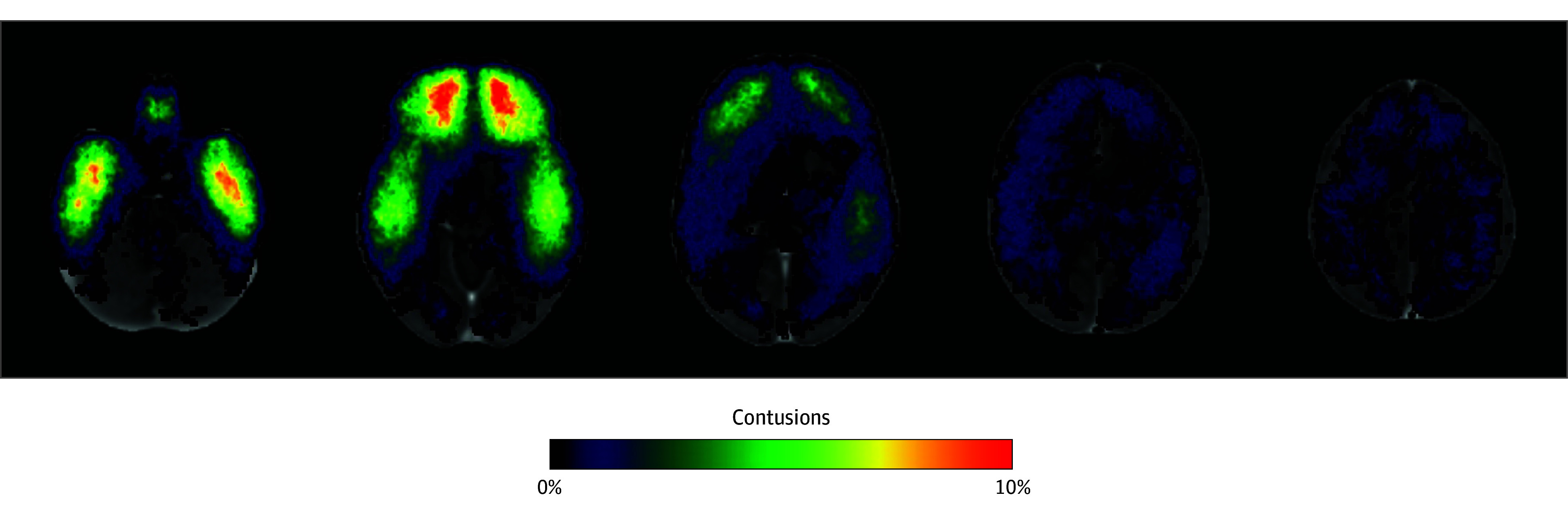
Regional Distribution of Contusions Contusions were registered to a common template and summed at each voxel. The color represents the proportion of the total Transforming Research and Clinical Knowledge in TBI cohort (N = 417) with a contusion at each voxel.

Contusions primarily affected the inferior frontal and temporal lobes (Figure 1). The regional distribution of the individual hyperdense (blood) and hypodense (edema) contusion components was similar (eFigure 4 in Supplement 1). The distribution of frontal and temporal contusion volumes were similar (75th percentile frontal, 2.20 cm^3^; 75th percentile temporal, 2.07 cm^3^) (Table 2), but a higher, nonsignificant proportion of outlier (>1.5 IQR above the 75th percentile) temporal contusion volumes was observed (15.6% vs 9.8%, respectively; McNemar *P* = .06).

**Figure 2.**
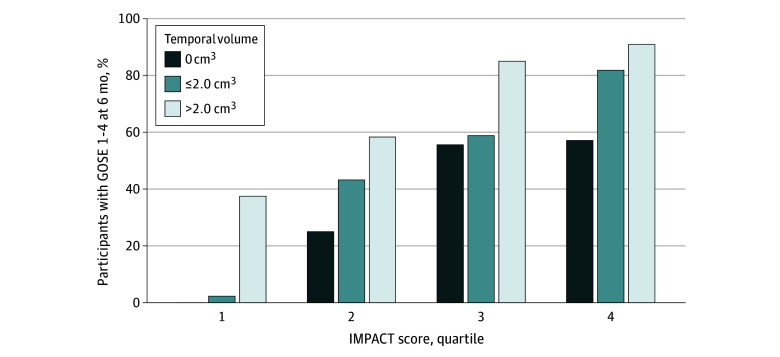
Temporal Contusion Volumes and Probability of Unfavorable Outcome Across IMPACT Quartiles GOSE 1-4 indicates Glasgow Outcome Scale-Extended, with scores of 1 to 4 indicating unfavorable outcomes at 6 months; IMPACT, International Mission for Prognosis and Analysis of Clinical Trials in TBI.

Contusion volumes in the frontal and temporal lobes were independently associated with unfavorable outcomes (frontal ≤2 cm^3^ vs absent: adjusted odds ratio [AOR], 2.65 [95% CI, 1.15-6.11]; frontal >2 cm^3^ vs absent: AOR, 3.38 [95% CI, 1.28-8.90]; temporal ≤2 cm^3^ vs absent: AOR, 1.82 [95% CI, 0.89-3.75]; temporal >2 cm^3^ vs absent: AOR, 4.88 [95% CI, 1.97-12.09]) (eTable 2 in Supplement 1 [model 4]). However, the discrimination of the IMPACT score was only improved significantly by the addition of temporal (AUROC, 0.86 vs 0.84; *P* = .03) (eTable 2 in Supplement 1 [model 3 vs model 1]) but not frontal (AUROC, 0.85 vs 0.84; *P* = .19) (eTable 2 in Supplement 1 [model 2 vs model 1]) contusion volumes. The addition of frontal contusion volumes did not change the discrimination of an IMPACT plus temporal contusion model (AUROC, 0.86 vs 0.86; *P* = .47) (eTable 2 in Supplement 1 [model 4 vs model 3]). Larger temporal contusion size was associated with an increased risk of unfavorable outcomes across IMPACT score quartiles (Figure 2). Comparing participants without temporal contusions with those in the largest volume quartile, we found that the incidence of unfavorable outcomes increased from 0% to 38% in the lowest IMPACT score quartile and from 57% to 91% in the highest IMPACT quartile (Figure 2). These results were similar in the analysis of hyperdense (eTable 3 and eFigure 5 in Supplement 1) and hypodense (eTable 4 in Supplement 1) contusion components separately for the secondary outcome measure (GOSE score ≤3) (eTable 5 in Supplement 1) or after excluding participants in the lowest contusion volume quartile (eTable 6 in Supplement 1).

**Figure 3.**
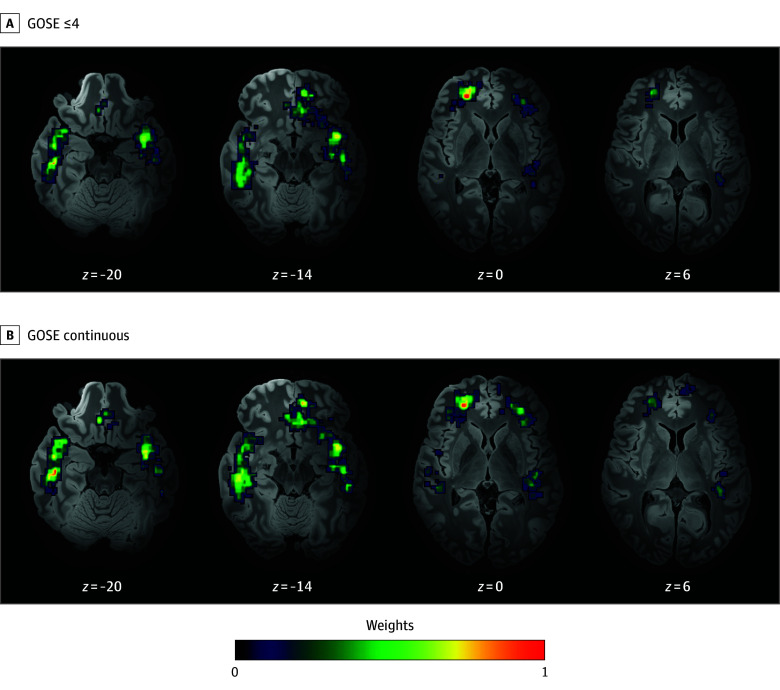
Disability Heat Map Heat maps were generated using 2 different outcomes: Glasgow Outcome Scale–Extended (GOSE) score of 4 or less at 6 months (A) and using the GOSE score as a continuous measure (B). Sparse canonical correlation analysis was used to identify the strongest independent voxelwise associations between the presence of a contusion and outcomes in the total International Mission for Prognosis and Analysis of Clinical Trials in TBI cohort (N = 417). The color indicates each voxel’s weight, a normalized value representing the strength of the association between the presence of a contusion and an unfavorable outcome. The redder the color, the greater the likelihood of an unfavorable outcome. The size of each cluster of colors indicates the range of brain areas that a contusion can hit to influence the outcome. The resulting maps were registered to a high-resolution Montreal Neurological Institute template for visualization.

To identify the strongest brainwide associations between contusions and unfavorable outcomes in a data-driven manner, we performed a multivariate voxelwise analysis. The resulting disability heat map (N = 417; cross-validation *r* = 0.33; *P* < .001) (Figure 3A) had both medial frontal and temporal clusters but a larger cluster extent in the temporal lobes. A nearly identical map was generated when treating GOSE as a continuous outcome measure (cross-validation *r* = 0.35; *P* < .001) (Figure 3B).

## Discussion

In this large, multicenter, prospective prognostic cohort study of participants with moderate or severe TBI, we found that by adding an artificial intelligence–based contusion labeling algorithm^11^ to the IMPACT score, temporal lobe contusions have a greater independent prognostic value than frontal lobe contusions in patients. Contusions of the same size affecting different brain regions were not prognostically equivalent. A brainwide search for the strongest associations with unfavorable outcomes identified specific clusters in the temporal and, to a lesser extent, medial frontal lobes. Collectively, these results suggest that an automated contusion identification pipeline, applied without manual intervention to heterogeneous, clinically acquired CT scans, may improve the performance of the best-available prognostic model in moderate or severe TBI. This automated approach might help to standardize prognoses across hospitals.

The association between contusion volume and outcomes differed between the frontal and temporal lobes. The largest temporal contusions may carry a higher odds of death or dependency compared with similarly sized frontal contusions. Furthermore, only temporal contusion volume significantly improved the discriminative capacity of the IMPACT score. This discrepancy between frontal and temporal contusions may exist because temporal contusions produce more lateral shift in the temporal fossa or, perhaps, more lateral shift at the level of the pineal gland. Although the Marshall CT score (included in the IMPACT score) captures the shift at the level of the septum pellucidum,^10^ the degree of pineal shift—a better marker for level of consciousness^39^—may not be adequately represented. Alternatively, temporal contusions may produce greater disruption to language^40^ and memory circuits^41^ than similar volumes of frontal injury, leading to greater functional impairment. Along these lines, perhaps deficits in executive function resulting from frontal contusions are less likely to result in lower scores on global outcome scales. However, we cannot fully exclude the possibility that at least part of the observed discrepancy was due to the largest temporal contusions being slightly larger than the largest frontal contusions.

The odds ratio estimate of the small temporal contusion volume (0-2 cm^3^) for unfavorable outcome was not significant in the primary model, suggesting that perhaps only the largest quartile of temporal contusions are prognostically relevant after accounting for the IMPACT score. However, the odds ratio remained close to 2 and was significant in the sensitivity analyses looking strictly at the hyperdense contusion component (eTable 3 in Supplement 1), using a secondary outcome measure (GOSE score ≤3) (eTable 5 in Supplement 1), and after excluding the smallest quartile of contusions (eTable 6 in Supplement 1).

Our data-driven disability heat map, while confirming a larger cluster extent in the temporal lobes, had 2 medial frontal clusters abutting the genu of the corpus callosum. Injury to the corpus callosum,^42,43^ particularly the genu,^44^ has been associated with cognitive impairment after TBI. Contusions that disrupt white matter fibers crossing the anterior callosum may lead to worse cognitive and functional outcomes.

Magnetic resonance imaging (MRI) offers substantially better tissue resolution than CT and may enable more accurate lesion border delineation. Studies have suggested that MRI-based approaches to improving prognostic models may be promising,^45^ but it remains unclear whether MRI is better than a clinical assessment^46^ at the time of imaging. Recent comprehensive lesion symptom studies were not able to explain any additional variance in outcomes with imaging when postinjury behavioral data are included as covariates.^47^ Additionally, MRI-based TBI studies frequently require highly selected samples with images acquired at some delay (often up to 1 month) from the initial injury, limiting the generalizability and clinical utility of the findings. The barriers to acquiring and analyzing CT data are lower, as are the barriers to clinically translating relevant findings.

The net prognostic benefit observed in this study was small. At this stage, it is premature to consider incorporation of this technique into clinical workflows. However, it is important to emphasize that we used clinically acquired scans and adjusted for a full complement of known prognostic indicators, including acute behavioral data. Furthermore, most contusions in this study were small (median total volume of 2.4 cm^3^), leaving room for other types of pathology, such as axonal injury, to have a more dominant influence on outcomes.

### Limitations

Several limitations of the dataset, the BLAST-CT algorithm, and the analysis are important to consider. First, CT scans within TRACK-TBI are not acquired using a standard protocol or at a standard time after injury. Compared with a more uniform sample, these factors add variability but increase the clinical generalizability of the findings. Second, we did not manually curate the label maps generated by BLAST-CT. The boundaries of each lesion may differ from those drawn by an expert human tracer. However, in a mixed-density contusion, the ground truth label on a CT scan is ultimately unclear. While our lack of curation may reduce the anatomic precision of our analyses, it provides a more direct assessment of the clinical utility of this tool. Third, we excluded participants with missing data. There were some minor but important differences between patients who were or were not missing GOSE and IMPACT scores, which could bias the results in unpredictable ways. Fourth, CT scans are insensitive to certain types of neuropathology (eg, axonal injury, which may account for much of the variance in disability outcomes that we observed). Fifth, the 6-month end point may be premature for determining a patient’s longer-term functional status after a severe brain injury. While there is minimal change in the distribution between 6-month and 1-year GOSE scores in the TRACK-TBI dataset,^14^ studies including only survivors have suggested improvement that extends beyond the 6-month time window.^48,49,50,51^ Finally, the net prognostic benefit in this study was small. A global disability measure, such as the GOSE, may not be well suited to detecting differential performance across cognitive domains, where the effects of frontal contusions may be more apparent.

## Conclusions

In this large, prospective prognostic cohort study of participants with moderate or severe TBI, use of an open-source, artificial intelligence–based measurement tool revealed that adding temporal, but not frontal, contusion volumes was associated with improved performance of the IMPACT score. Understanding why such regional differences exist may shed light on the neuroanatomic mechanisms of disability after TBI, helping to stratify patients for trials and identify neuromodulatory treatment targets. The disability heat map that we identified represents an exciting area for future investigation.

## Notes

Supplement 1. **eTable 1.** Cohort Characteristics by Missingness **eTable 2.** Logistic Regression Models for Primary Outcome **eTable 3.** Logistic Regression Models for Hyperdense Contusion Component (Hemorrhage) **eTable 4.** Logistic Regression Models for Hypodense Contusion Component (Edema) **eTable 5.** Logistic Regression Models for Secondary Outcome (GOSE ≤3) **eTable 6.** Logistic Regression Models for Primary Outcome, Excluding Lowest Contusion Volume Quartile Participants (N = 219) **eFigure 1.** BLAST-CT Validation **eFigure 2.** Study Flowchart **eFigure 3.** Six-Month Outcomes **eFigure 4.** Regional Distribution of Hyperdense vs Hypodense Contusion Components **eFigure 5.** ROC Curves for the Addition of Regional Contusion Volumes to the IMPACT Score

Supplement 2.
Data Sharing Statement
