# Supplementary material for: Automated Measurement of Cerebral Hemorrhagic Contusions and Outcomes After Traumatic Brain Injury in the TRACK-TBI Study
Source: JAMA Netw Open. 2024 Aug 30;7(8):e2427772. doi: 10.1001/jamanetworkopen.2024.27772 (PMC11365003; doi:10.1001/jamanetworkopen.2024.27772)
Supplement: Supplement 1. — eTable 1. Cohort Characteristics by Missingness eTable 2. Logistic Regression Models for Primary Outcome eTable 3. Logistic Regression Models for Hyperdense Contusion Component (Hemorrhage) eTable 4. Logistic Regression Models for Hypodense Contusion Component (Edema) eTable 5. Logistic Regression Models for Secondary Outcome (GOSE ≤3) eTable 6. Logistic Regression Models for Primary Outcome, Excluding Lowest Contusion Volume Quartile Participants (N = 219) eFigure 1. BLAST-CT Validation eFigure 2. Study Flowchart eFigure 3. Six-Month Outcomes eFigure 4. Regional Distribution of Hyperdense vs Hypodense Contusion Components eFigure 5. ROC Curves for the Addition of Regional Contusion Volumes to the IMPACT Score [file jamanetwopen-e2427772-s001.pdf]

Supplemental Online Content

Snider SB, Temkin NR, Sun X, et al; TRACK-TBI Investigators. Automated measurement of cerebral hemorrhagic contusions and outcomes after traumatic brain injury in the TRACK-TBI study. *JAMA Netw Open*. 2024;7(8):e2427772. doi:10.1001/jamanetworkopen.2024.27772

eTable 1. Cohort Characteristics by Missingness

eTable 2. Logistic Regression Models for Primary Outcome

eTable 3. Logistic Regression Models for Hyperdense Contusion Component (Hemorrhage)

eTable 4. Logistic Regression Models for Hypodense Contusion Component (Edema)

eTable 5. Logistic Regression Models for Secondary Outcome (GOSE ≤3)

eTable 6. Logistic Regression Models for Primary Outcome, Excluding Lowest Contusion Volume Quartile Participants (N = 219)

eFigure 1. BLAST-CT Validation

eFigure 2. Study Flowchart

eFigure 3. Six-Month Outcomes

eFigure 4. Regional Distribution of Hyperdense vs Hypodense Contusion Components

eFigure 5. ROC Curves for the Addition of Regional Contusion Volumes to the IMPACT Score

This supplemental material has been provided by the authors to give readers additional information about their work.

© 2024 Snider SB et al. *JAMA Network Open.*

**eTable 1.** Cohort Characteristics by Missingness						
	**IMPACT Score**			**GOSE Score**		
	Available	Missing	*P value*	Available	Missing	*P value*
**Age**<br>Mean (SD)	42 (18)	44 (19)	0.27	42 (18)	38 (16)	0.024
**Sex**			0.53			0.49
Male	221 (76%)	100 (79%)		321 (77%)	116 (80%)	
Female	79 (24%)	26 (21%)		96 (23%)	29 (20%)	
Total	291 (100%)	126 (100%)		417 (100%)	145 (100%)	
**Race**			0.75			0.032
White	231 (81%)	100 (84%)		331 (82%)	100 (71%)	
Black	37 (13%)	12 (10%)		49 (12%)	27 (19%)	
Other	17 (6%)	7 (6%)		24 (6%)	13 (9%)	
Total	285 (100%)	119 (100%)		404 (100%)	140 (100%)	
**Ethnicity**			0.89			0.08
Non-Hispanic	236 (83%)	100 (83%)		336 (83%)	107 (76%)	
Hispanic	50 (17%)	20 (17%)		70 (17%)	34 (24%)	
Total	286 (100%)	120 (100%)		406 (100%)	141 (100%)	
**Years Education**<br>Mean (SD)	13 (2)	13 (2)	0.49	13 (2)	12 (3)	0.11
**Disposition**			1.0			0.033
ED Discharge	1 (0%)	0 (0%)		1 (0%)	1 (0%)	
Hospital admit - ICU	4 (2%)	1 (1%)		5 (1%)	6 (4%)	
Hospital admit + ICU	286 (98%)	125 (99%)		411 (99%)	138 (95%)	
Total	291 (100%)	126 (100%)		417 (100%)	141 (100%)	
**Injury Cause**			0.72			0.015
Road traffic incident	165 (57%)	71 (57%)		236 (57%)	84 (58%)	
Incidental fall	81 (28%)	39 (31%)		120 (29%)	27 (19%)	
Violence/assault	17 (6%)	8 (6%)		25 (6%)	19 (13%)	
Other	25 (9%)	7 (6%)		32 (8%)	14 (10%)	
Total	288 (100%)	125 (100%)		413 (100%)	144 (100%)	
**Psychiatric History**			0.90			0.25
No	222 (76%)	97 (77%)		319 (77%)	118 (81%)	
Yes	69 (24%)	29 (23%)		98 (24%)	27 (19%)	
Total	291 (100%)	126 (100%)		417 (100%)	145 (100%)	
**Prior TBI**			0.30			0.76
No	208 (84%)	77 (89%)		285 (85%)	98 (87%)	
Yes	41 (16%)	10 (11%)		51 (15%)	15 (13%)	
Total	249 (100%)	87 (100%)		336 (100%)	113 (100%)	
**GCS ED Arrival**						
13-15	36 (13%)	13 (12%)	0.94	49 (13%)	36 (26%)	< 0.001
9-12	54 (19%)	22 (21%)		76 (20%)	34 (25%)	
3-8	191 (68%)	71 (67%)		262 (68%)	67 (49%)	
Total	281 (100%)	106 (100%)		387 (100%)	137 (100%)	
Median (Q1, Q3)	7 (3, 10)	7 (3, 9)	0.73	7 (3, 10)	8 (3, 13)	< 0.001

© 2024 Snider SB et al. *JAMA Network Open.*

**eTable 2.** Logistic Regression Models for Primary Outcome		
	**aOR (95% CI)**	**P value**
**MODEL 1**		
IMPACT$_{core+CT}$ score*	1.84 (1.60, 2.11)	<0.001
McFadden's Pseudo-R^2=0.28, Nagelkerke's=0.42, AUC=0.836		
**MODEL 2**		
IMPACT$_{core+CT}$ score	1.82 (1.58, 2.10)	<0.001
Frontal Vol. absent<br>Frontal Vol. (≤ 2cc)<br>Frontal Vol. (> 2cc)	1<br>2.63 (1.17, 5.94)<br>4.61 (1.83,11.65)	0.005
McFadden's Pseudo-R^2=0.30, Nagelkerke's=0.46, AUC=0.848 (p=0.19 vs Model 1)		
**MODEL 3**		
IMPACT$_{core+CT}$ score	1.78 (1.54, 2.05)	<0.001
Temporal Vol. absent<br>Temporal Vol. (≤ 2cc)<br>Temporal Vol. (> 2cc)	1<br>1.67 (0.83, 3.39)<br>5.46 (2.28, 13.07)	< 0.001
McFadden's Pseudo-R^2=0.32, Nagelkerke's=0.47, AUC=0.857 (p=0.031 vs Model 1)		
**MODEL 4**		
IMPACT$_{core+CT}$ score	1.77 (1.53, 2.04)	<0.001
Frontal Vol. absent<br>Frontal Vol. (≤ 2cc)<br>Frontal Vol. (> 2cc)	1<br>2.65 (1.15, 6.11)<br>3.38 (1.28, 8.90)	0.034
Temporal Vol. absent<br>Temporal Vol. (≤ 2cc)<br>Temporal Vol. (> 2cc)	1<br>1.82 (0.89, 3.75)<br>4.88 (1.97, 12.09)	0.003
McFadden's Pseudo-R^2=0.33, Nagelkerke's=0.49, AUC=0.862 (p=0.47 vs model 3)		

*aOR for IMPACT$_{core+CT}$ score reflects odds associated with a 10 point change in the predicted probability of unfavorable outcome. Abbreviations (aOR = adjusted Odds Ratio, cc = cubic centimeter, Vol = Volume, GOSE = Glasgow Outcome Scale Extended)

© 2024 Snider SB et al. *JAMA Network Open.*

**eTable 3.** Logistic Regression Models for Hyperdense Contusion Component (Hemorrhage)		
	**aOR (95% CI)**	**P value**
**MODEL 1**		
IMPACT$_{core+CT}$ score*	1.84 (1.60, 2.11)	<0.001
McFadden's Pseudo-R^2=0.28, Nagelkerke's=0.42, AUC=0.836		
**MODEL 5A**		
IMPACT$_{core+CT}$ score	1.82 (1.58, 2.09)	<0.001
Frontal Vol. absent<br>Frontal Vol. (≤ 1cc)<br>Frontal Vol. (> 1cc)	1<br>2.00 (1.03, 3.89)<br>2.90 (1.32, 6.34)	0.021
McFadden's Pseudo-R^2=0.30, Nagelkerke's=0.45, AUC=0.844 (p=0.27 vs Model 1)		
**MODEL 6A**		
IMPACT$_{core+CT}$ score	1.80 (1.55, 2.08)	<0.001
Temporal Vol. absent<br>Temporal Vol. (≤ 1cc)<br>Temporal Vol. (> 1cc)	1<br>2.86 (1.47, 5.56)<br>5.32 (2.39, 11.85)	< 0.001
McFadden's Pseudo-R^2=0.33, Nagelkerke's=0.49, AUC=0.86 (p=0.028 vs Model 1)		
**MODEL 7A**		
IMPACT$_{core+CT}$ score	1.79 (1.54, 2.07)	<0.001
Frontal Vol. absent<br>Frontal Vol. (≤ 1cc)<br>Frontal Vol. (> 1cc)	1<br>1.92 (0.96, 3.85)<br>2.13 (0.92, 4.93)	0.11
Temporal Vol. absent<br>Temporal Vol. (≤ 1cc)<br>Temporal Vol. (> 1cc)	1<br>2.87 (1.46, 5.61)<br>4.60 (2.00, 10.58)	< 0.001
McFadden's Pseudo-R^2=0.34, Nagelkerke's=0.50, AUC=0.864 (p=0.48 vs model 6A)		

*aOR for IMPACT$_{core+CT}$ score reflects odds associated with a 10 point change in the predicted probability of unfavorable outcome. Abbreviations (aOR = adjusted Odds Ratio, cc = cubic centimeter, Vol = Volume, GOSE = Glasgow Outcome Scale Extended)

© 2024 Snider SB et al. *JAMA Network Open.*

**eTable 4.** Logistic Regression Models for Hypodense Contusion Component (Edema)		
	**aOR (95% CI)**	**P value**
**MODEL 1**		
IMPACT$_{core+CT}$ score*	1.84 (1.60, 2.11)	<0.001
McFadden's Pseudo-R^2=0.28, Nagelkerke's=0.42, AUC=0.836		
**MODEL 5B**		
IMPACT$_{core+CT}$ score	1.81 (1.57, 2.08)	<0.001
Frontal Vol. absent<br>Frontal Vol. (≤ 1cc)<br>Frontal Vol. (> 1cc)	1<br>1.94 (0.97, 3.90)<br>2.83 (1.25, 6.41)	0.039
McFadden's Pseudo-R^2=0.29, Nagelkerke's=0.44, AUC=0.844 (p=0.25 vs Model 1)		
**MODEL 6B**		
IMPACT$_{core+CT}$ score	1.78 (1.53, 2.05)	<0.001
Temporal Vol. absent<br>Temporal Vol. (≤ 1cc)<br>Temporal Vol. (> 1cc)	1<br>1.21 (0.62, 2.37)<br>4.31 (1.89, 9.81)	0.001
McFadden's Pseudo-R^2=0.31, Nagelkerke's=0.47, AUC=0.854 (p=0.055 vs Model 1)		
**MODEL 7B**		
IMPACT$_{core+CT}$ score	1.76 (1.53, 2.04)	<0.001
Frontal Vol. absent<br>Frontal Vol. (≤ 1cc)<br>Frontal Vol. (> 1cc)	1<br>1.78 (0.88, 3.62)<br>1.82 (0.76, 4.36)	0.24
Temporal Vol. absent<br>Temporal Vol. (≤ 1cc)<br>Temporal Vol. (> 1cc)	1<br>1.19 (0.60, 2.34)<br>3.83 (1.62, 9.07)	0.005
McFadden's Pseudo-R^2=0.32, Nagelkerke's=0.48, AUC=0.857 (p=0.47 vs model 6B)		

*aOR for IMPACT$_{core+CT}$ score reflects odds associated with a 10 point change in the predicted probability of unfavorable outcome. Abbreviations (aOR = adjusted Odds Ratio, cc = cubic centimeter, Vol = Volume, GOSE = Glasgow Outcome Scale Extended)

© 2024 Snider SB et al. *JAMA Network Open.*

**eTable 5.** Logistic Regression Models for Secondary Outcome (GOSE ≤3)		
	**aOR (95% CI)**	**P value**
**MODEL 7**		
IMPACT$_{core+CT}$ score*	1.84 (1.60, 2.12)	<0.001
McFadden's Pseudo-R^2=0.28, Nagelkerke's=0.42, AUC=0.837		
**MODEL 8**		
IMPACT$_{core+CT}$ score	1.83 (1.58, 2.11)	<0.001
Frontal Vol. absent<br>Frontal Vol. (≤ 2cc)<br>Frontal Vol. (> 2cc)	1<br>2.03 (0.90, 4.59)<br>4.62 (1.83,11.69)	0.004
McFadden's Pseudo-R^2=0.31, Nagelkerke's=0.46, AUC=0.849 (p=0.17 vs Model 1)		
**MODEL 9**		
IMPACT$_{core+CT}$ score	1.78 (1.54, 2.06)	<0.001
Temporal Vol. absent<br>Temporal Vol. (≤ 2cc)<br>Temporal Vol. (> 2cc)	1<br>1.95 (0.94, 4.05)<br>6.48 (2.68, 15.65)	<0.001
McFadden's Pseudo-R^2=0.32, Nagelkerke's=0.48, AUC=0.861 (p=0.025 vs Model 1)		
**MODEL 10**		
IMPACT$_{core+CT}$ score	1.77 (1.53, 2.05)	<0.001
Frontal Vol. absent<br>Frontal Vol. (≤ 2cc)<br>Frontal Vol. (> 2cc)	1<br>2.04 (0.88, 4.71)<br>3.44 (1.30, 9.09)	0.045
Temporal Vol. absent<br>Temporal Vol. (≤ 2cc)<br>Temporal Vol. (> 2cc)	1<br>2.12 (1.00, 4.47)<br>5.45 (2.19, 13.52)	0.001
McFadden's Pseudo-R^2=0.34, Nagelkerke's=0.50, AUC=0.866 (p=0.45 vs model 3)		

*aOR for IMPACT$_{core+CT}$ score reflects odds associated with a 10 point change in the predicted probability of unfavorable outcome. Abbreviations (aOR = adjusted Odds Ratio, cc = cubic centimeter, Vol = Volume, GOSE = Glasgow Outcome Scale Extended)

© 2024 Snider SB et al. *JAMA Network Open.*

**eTable 6.** Logistic Regression Models for Primary Outcome, Excluding Lowest Contusion Volume Quartile Participants (N = 219)		
	**aOR (95% CI)**	**P value**
**MODEL 11**		
$IMPACT_{core+CT}$ score*	1.74 (1.49, 2.02)	<0.001
McFadden's Pseudo-R^2=0.23, Nagelkerke's=0.37, AUC=0.812		
**MODEL 12**		
$IMPACT_{core+CT}$ score	1.75 (1.49, 2.04)	<0.001
Frontal Vol. absent<br>Frontal Vol. (0.1 to 4.7 cc)<br>Frontal Vol. (> 4.7 cc)	1<br>1.25 (0.54, 2.89)<br>2.22 (0.79,6.25)	0.28
McFadden's Pseudo-R^2=0.24, Nagelkerke's=0.38, AUC=0.817 (p=0.43 vs Model 11)		
**MODEL 13**		
$IMPACT_{core+CT}$ score	1.72 (1.46, 2.02)	<0.001
Temporal Vol. absent<br>Temporal Vol. (0.1 to 4.7 cc)<br>Temporal Vol. (> 4.7 cc)	1<br>2.10 (0.98, 4.51)<br>6.71 (2.57, 17.48)	0.001
McFadden's Pseudo-R^2=0.29, Nagelkerke's=0.44, AUC=0.839 (p=0.061 vs Model 11)		
**MODEL 14**		
$IMPACT_{core+CT}$ score	1.72 (1.46, 2.03)	<0.001
Frontal Vol. absent<br>Frontal Vol. (0.1 to 4.7 cc)<br>Frontal Vol. (> 4.7 cc)	1<br>1.46 (0.60, 3.53)<br>2.07 (0.68, 6.29)	0.44
Temporal Vol. absent<br>Temporal Vol. (0.1 to 4.7 cc)<br>Temporal Vol. (> 4.7 cc)	1<br>2.30 (1.05, 5.04)<br>6.61 (2.49, 17.54)	0.001
McFadden's Pseudo-R^2=0.30, Nagelkerke's=0.45, AUC=0.843 (p=0.46 vs model 13)		

*aOR for $IMPACT_{core+CT}$ score reflects odds associated with a 10 point change in the predicted probability of unfavorable outcome. Abbreviations (aOR = adjusted Odds Ratio, cc = cubic centimeter, Vol = Volume, GOSE = Glasgow Outcome Scale Extended)

© 2024 Snider SB et al. *JAMA Network Open.*

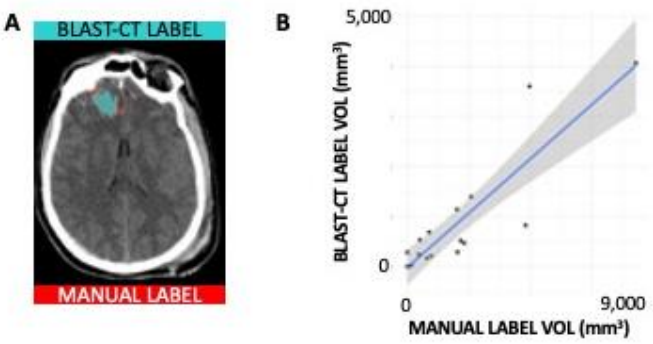

eFigure 1. BLAST-CT Validation

(A) A representative BLAST-CT contusion label (blue) is shown overlaid on a manual label (red). (B) In 18 randomly chosen subjects, manual label volumes were correlated with BLAST-CT label volumes.

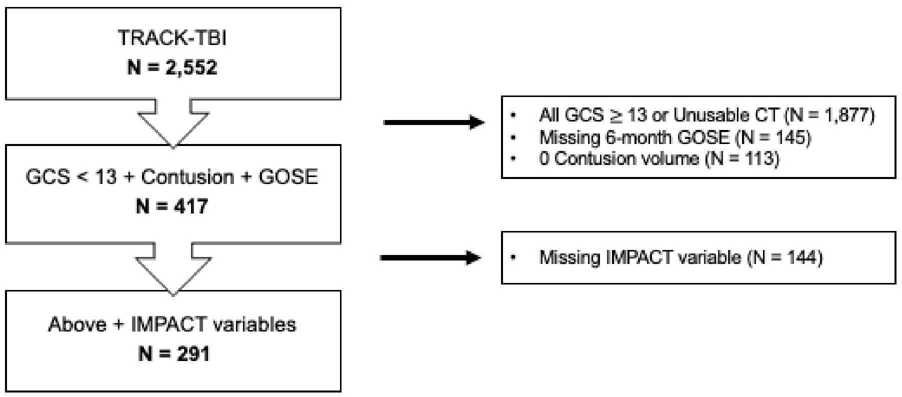

eFigure 2. Study Flowchart

Abbreviations: TRACK-TBI Transforming Research and Clinical Knowledge in Traumatic Brain Injury; IMPACT International Mission for Prognosis and Analysis of Clinical Trials; GOSE Glasgow Outcome Scale Extended; GCS Glasgow Coma Scale

© 2024 Snider SB et al. *JAMA Network Open.*

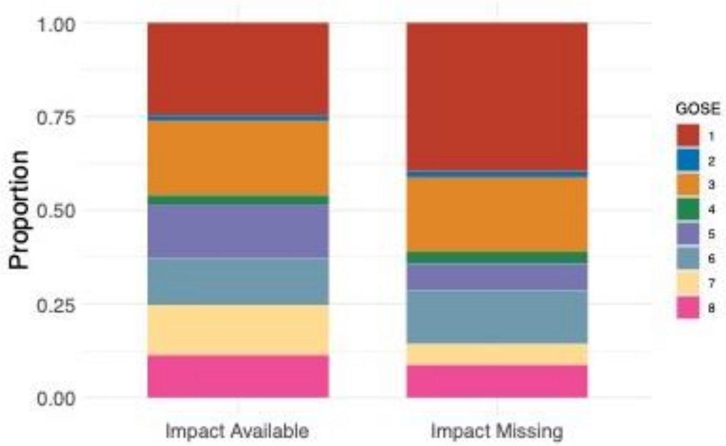

eFigure 3. Six-Month Outcomes
Distirbution of 6-month Glasgow Outcome Scale Extended (GOSE) outcomes shown for participants with all IMPACT covariates available (N=291) compared to any covariate missing (N=126).

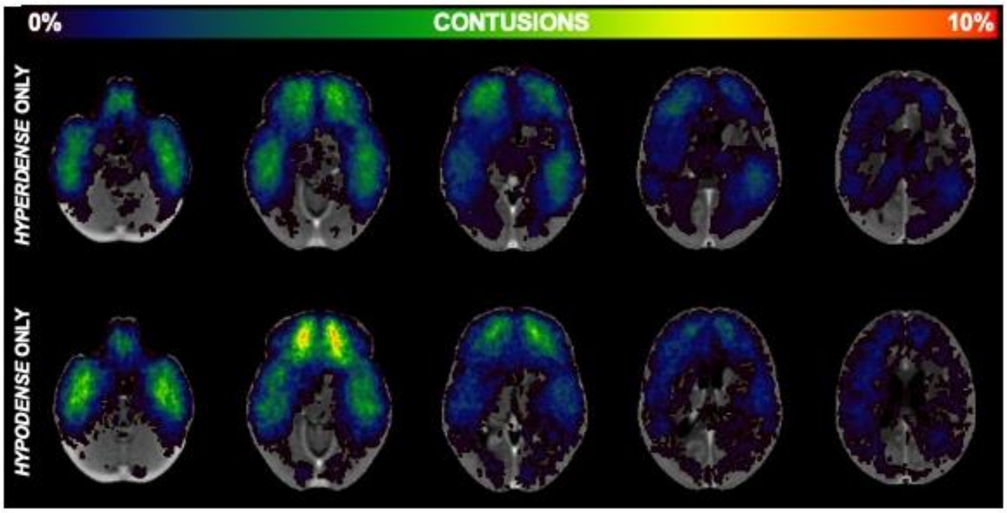

eFigure 4. Regional Distribution of Hyperdense vs Hypodense Contusion Components
The hyperdense (top row) and hypodense (bottom row) parenchymal labels from the BLAST-CT algorithm output were separately extracted, registered to a common template, and summed at each voxel. The color represents the proportion (0-10%) of the total cohort (N=417) with a lesion at each voxel.

© 2024 Snider SB et al. *JAMA Network Open.*

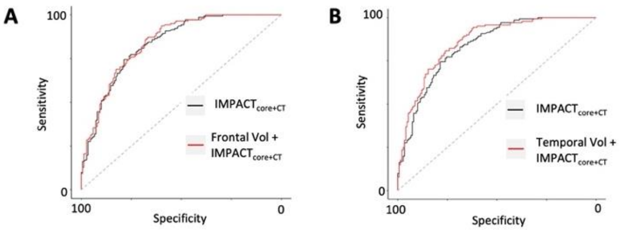

eFigure 5. ROC Curves for the Addition of Regional Contusion Volumes to the IMPACT Score

ROC Curves for $IMPACT_{core+CT}$ score alone (black) and $IMPACT_{core+CT}$ + Frontal hyperdense contusion component volume (red, A) or $IMPACT_{core+CT}$ + Temporal hyperdense contusion component volume (red, B).

© 2024 Snider SB et al. *JAMA Network Open.*
