# Supplementary material for: Automated Measurement of Cerebral Hemorrhagic Contusions and Outcomes After Traumatic Brain Injury in the TRACK-TBI Study
Source: JAMA Netw Open. 2024 Aug 30;7(8):e2427772. doi: 10.1001/jamanetworkopen.2024.27772 (PMC11365003; doi:10.1001/jamanetworkopen.2024.27772)
Supplement: Supplement 2. — Data Sharing Statement [file jamanetwopen-e2427772-s002.pdf]

Data Sharing Statement

Snider. Automated Measurement of Cerebral Hemorrhagic Contusions and Outcomes After Traumatic Brain Injury in the TRACK-TBI Study. *JAMA Netw Open*. Published August 30, 2024. doi:10.1001/jamanetworkopen.2024.27772

Data
Data available: No

Additional Information
Explanation for why data not available: Available on request to central committee
